# Quercetin Enhances the Antitumor Activity of Trichostatin A through Upregulation of p53 Protein Expression In Vitro and In Vivo

**DOI:** 10.1371/journal.pone.0054255

**Published:** 2013-01-16

**Authors:** Shu-Ting Chan, Nae-Cherng Yang, Chin-Shiu Huang, Jiunn-Wang Liao, Shu-Lan Yeh

**Affiliations:** 1 Department of Nutritional Science, Chung Shan Medical University, Taichung, Taiwan; 2 Department of Health and Nutrition Biotechnology, Asia University, Taichung, Taiwan; 3 Graduate Institute of Veterinary Pathology, College of Veterinary Medicine, National Chung Hsing University, Taichung, Taiwan; 4 Department of Nutrition, Chung Shan Medical University Hospital, Taichung, Taiwan; Virginia Commonwealth University, United States of America

## Abstract

This study investigated the effects of quercetin on the anti-tumor effect of trichostatin A (TSA), a novel anticancer drug, in vitro and in vivo and the possible mechanisms of these effects in human lung cancer cells. We first showed that quercetin (5 µM) significantly increased the growth arrest and apoptosis in A549 cells (expressing wild-type p53) induced by 25 ng/mL of (82.5 nM) TSA at 48 h by about 25% and 101%, respectively. However, such enhancing effects of quercetin (5 µM) were not significant in TSA-exposed H1299 cells (a p53 null mutant) or were much lower than in A549 cells. In addition, quercetin significantly increased TSA-induced p53 expression in A549 cells. Transfection of p53 siRNA into A549 cells significantly but not completely diminished the enhancing effects of quercetin on TSA-induced apoptosis. Furthermore, we demonstrated that quercetin enhanced TSA-induced apoptosis through the mitochondrial pathway. Transfection of p53 siRNA abolished such enhancing effects of quercetin. However, quercetin increased the acetylation of histones H3 and H4 induced by TSA in A549 cells, even with p53 siRNA transfection as well as in H1299 cells. In a xenograft mouse model of lung cancer, quercetin enhanced the antitumor effect of TSA. Tumors from mice treated with TSA in combination with quercetin had higher p53 and apoptosis levels than did those from control and TSA-treated mice. These data indicate that regulation of the expression of p53 by quercetin plays an important role in enhancing TSA-induced apoptosis in A549 cells. However, p53-independent mechanisms may also contribute to the enhancing effect of quercetin.

## Introduction

Trichostatin A (TSA) is a histone deacetylase inhibitor, which is a member of the promising class of anti-cancer drugs that selectively induce the differentiation and apoptosis of various transformed cells [1]. The accumulation of acetylated histones and nonhistone proteins is an important mechanism by which TSA affects the transcriptional patterns of many genes, including those associated with cell growth arrest and apoptosis [2], [3]. Several studies have suggested that TSA may be a potential therapy for lung cancer [4]–[7] because it induces apoptosis of lung cancer cells by activating the death receptor and mitochondria-mediated pathways [4]. However, the toxicity of this drug, such as the cardiac hypertrophy effect, limits its application [8].

Several phytochemicals, such as quercetin, have been reported to prevent cancer development by themselves or by enhancing the effects of anti-cancer drugs [9]–[12]. Quercetin, a flavonoid, is ubiquitously found in various vegetarian foods, and research suggests it may act to prevent the development of cancers [13], [14]. Quercetin may exert its anti-cancer effect through several mechanisms, including acting as an antioxidant, inducing apoptosis, acting as an anti-inflammatory agent, and modulating signaling pathways [15]–[18]. For example, a recent study showed that quercetin at a concentration of 80 µM induces mitochondria-mediated apoptosis in HeLa cells through the activation of p53 [17]. In addition, it has been demonstrated that quercetin significantly increases the anti-cancer effect of doxorubicin in breast cancer cells and reduces the cytotoxic side effects of doxorubicin in non-tumor cells [19]. Chen and Kang [20] found that quercetin (10–40 µM) in combination with TSA cooperatively induces cell death in human leukemia HL-60 cells. However, the combined effect of TSA and quercetin in human lung cancer cells is unclear. We hypothesized that quercetin would be an effective adjuvant to TSA treatment in lung cancer cells.

Thus, the aim of this study was to investigate the enhancing effects of quercetin on the antitumor effect of TSA in vitro and in vivo. In addition, we investigated the role of p53 in the enhancing effect of quercetin because it has been reported that quercetin exerts its antitumor effect through upregulation of p53 expression [21]. We used two human lung cancer cell lines, A549 and H1299, to address this issue. A549 cells express wild-type p53 protein, whereas H1299 cells are a p53 null mutant. Moreover, we used a xenograft mouse model to confirm the in vitro findings.

## Materials and Methods

### Ethics Statement

Animal care followed the guidelines of the National Research Council and all study protocols were approved by the Institutional Animal Care and Use Committee at Chung Shan Medical University.

### Reagents

All chemicals used were reagent grade or higher. Quercetin and TSA were purchased from Sigma Chemical Co. (St. Louis, MO, USA). RPMI medium 1640, fetal bovine serum, trypsin, penicillin, streptomycin, sodium pyruvate, and nonessential amino acids were purchased from GIBCO/BRL (Rockville, MD, USA).

### Cell Culture and Cell Growth Test

A549 cells and H1299 cells were obtained from the American Type Culture Collection (Rockville, MD, USA). Both cell lines were cultured in RPMI medium 1640 containing 10% (v/v) FBS, 0.37% (w/v) NaHCO_3_, penicillin (100 units/mL), and streptomycin (100 µg/mL) at 37°C in a humidified incubator under 5% CO_2_ and 95% air. An equal number (2.5×10^4^/mL) of cells was incubated for 24 h before the various treatments. After being washed twice with PBS (pH 7.4, containing 137 mM NaCl, 2.7 mM KCl, 1.5 mM KH_2_PO_4_, and 8.1 mM Na_2_HPO_4_), the cells were incubated in fresh culture medium containing TSA (25 ng/mL equal to 82.5 nM) alone or in combination with quercetin. Stock solutions of ethanol-TSA (100 µg/mL) and ethanol-quercetin (20 mM) were freshly prepared before each experiment. The final solvent concentration in medium was ≦0.15%. The medium was replaced every day. Cell growth was mainly measured by MTT colorimetric assay.

### Annexin V-FITC-propidium Iodide Assay

An Annexin V-FITC apoptosis detection kit (BD Pharmingen, San Diego, CA, USA) was used to determine the number of apoptotic cells. According to the manufacturer’s instructions, the treated cells were harvested after the indicated time, washed twice with ice-cold PBS and resuspended in 100 µL of binding buffer. Then an aqueous mixture of Annexin V-FITC and propidium iodide staining buffer was added and the mixture was incubated in the dark at 37°C for 15 min. Before flow cytometric analysis, 400 µL of binding buffer was added to each sample. A total of 100,000 events per sample were analyzed. Flow cytometric analysis was performed with a FACS Calibur flow cytometer (BD Biosciences, Franklin Lakes, NJ, USA) with WinMDI 2.8 software.

### Western Blot Assay

The treated cells were harvested and lysed with 20% SDS containing 1 mM phenylmethyl sulfonyl fluoride. The lysate was sonicated for 1 min on ice followed by centrifugation at 12,000×*g* for 30 min at 4°C. Mitochondrial and cytosolic fractions were isolated by using the ProteoExtract® Cytosol/Mitochondria Fractionation Kit (Merck Millipore, Billerica, MA, USA). Then an amount of protein from the supernatant was resolved by SDS-PAGE and transferred onto a nitrocellulose membrane. After blocking with TBS buffer (20 mM Tris–HCl, 150 mM NaCl, pH 7.4) containing 5% nonfat milk, the membrane was incubated with antibodies against p53 (Santa Cruz Biotechnology, Santa Cruz, CA, USA), Bax, Apaf-1, Bcl2 (Gene Tex, CA, USA ), cytochrome c (Santa Cruz Biotechnology, Santa Cruz, CA, USA), or acetyl histones H3/H4 (Upstate Biotechnology, Lake Placid, NY, USA) followed by horseradish peroxidase–conjugated secondary antibodies and then was visualized with an ECL chemiluminescence detection kit (PerkinElmer Life Sciences, Waltham, MA, USA). The relative density of the immunoreactive bands was quantified by using a luminescent image analyzer (LSA-100, Fujifilm, Japan).

### Caspase-3 and Caspase-9 Activities

Caspase-3 and caspase-9 activities were measured by using colorimetric protease assay kits (Chemicon, Billerica, MA, USA) according to the manufacturer’s instructions. Protein concentrations of lysates were determined by the Lowry method [22]. An aliquot of cell lysates (70 µL) was incubated with the substrate of caspase-3 or caspase-9 at 37°C for 2 h. Samples were analyzed at 405 nm in a microplate reader (Molecular Devices, Sunnyvale, CA, USA). The relative caspase activity of the control group was taken as 100.

### Transfection of siRNA

A549 cells were transfected with 100 nM predesigned siRNA for *p53* (NM_000546; sequence: forward, 5′-GACUCCAGUGGUAAUCUACTT-3′; reverse, 5′-GUAGAUUACCACUGGAGUCTT-3′) by using LipofectAMINE 2000 (Invitrogen, Carlsbad, CA, USA). After 24 h of incubation, a fresh medium containing test compounds was added for another 24 h. Nontargeted siRNA was used as a negative control.

### Tumor Cell Xenograft Mouse Model

Thirty male nude mice (4 to 6 weeks of age) were obtained from the National Laboratory Animal Center (Taipei, Taiwan). The animals were housed in specific pathogen-free conditions with an alternating 12-hour light:dark cycle. After being acclimated for 1 week, the animals were subcutaneously injected in the right flank with A549 cells at a dose of 5×10^6^ cells (in 200 µL of matrigel; BD Biosciences, Franklin Lakes, NJ, USA). The tumor nodule volumes were measured once a week with the formula: (L1×L2^2^)/2 [23], where L1 is the long axis and L2 is the short axis of the tumor. Four weeks after cell injection, tumor nodules were palpable. The animals were then randomly assigned to the following five groups (n = 6/group) for 6 months: control group, low-dose TSA group, high-dose TSA group, quercetin alone group, and low-dose TSA+quercetin group. The mean tumor volume was not significantly different among the groups at this time. TSA was given by intraperitoneal injection at a dose of 0.5 mg/kg body weight (low-dose TSA) or 1 mg/kg body weight (high-dose TSA) twice a week. We chose these two doses of TSA because in our preliminary study we found that these doses showed no apparent toxicity in nude mice, whereas treatment with 2 mg/kg TSA for 4 weeks led to 4/5 animal deaths (data not shown). According to a previous study [24], quercetin was also given by intraperitoneal injection at a dose of 10 mg/kg body weight 3 times/week. Stock solutions of ethanol-TSA and ethanol-quercetin were freshly prepared before each injection and were diluted by 0.9% saline solution. The injection volume was 200 µL (containing 25 µL of stock solution and 175 µL 0.9% saline solution) each time. The control group was given 200 µL of vehicle (25 µL ethanol and 175 µL 0.9% saline solution) only. All animals were allowed free access to a standard rodent diet (Lab 5001, Purina Mills, St. Louis, MO) and water during the study. During the 4-month experimental period, the body weights of the mice were recorded weekly. There were no significant differences in body weight among the groups in the study (data not shown).

### Expression of p53 and Apoptosis in Tumor Tissue

After the mice were sacrificed, tumors were excised to determine p53 expression and apoptosis. Half of the tumor tissue from each mouse was stored in 10% formalin and the other half was stored at −80°C. Then the tissues stored in formalin were embedded in paraffin, sectioned, and subjected to immunohistochemical staining for p53 expression using the streptavidin-peroxidase technique as described previously [25]. The frozen tumor tissues (0.03 g) were homogenized with 300 µL lysis buffer (10% Trion-100, 0.1% SDS, 0.5% sodium deoxycholate) containing 1 mM phenylmethyl sulfonyl fluoride and centrifuged at 12,000×*g* for 30 min at 4°C. Then protein (250 µg) from the supernatant was used for p53 Western blot assay according the method described above. In addition, the levels of apoptosis of tumor tissue stored in formalin were detected using the Terminal Transferase dUTP Nick End Labeling (TUNEL) system following the manufacturer’s protocol (Chemicon, Billerica, MA, USA). The assay detects DNA fragmentation by terminal deoxynucleotidyl transferase. The enzyme catalyzes the addition of dUTPs that are secondarily labeled with a marker. The images of TUNEL and immunohistochemical staining were determined by microscopy at ×400 magnification.

### Statistical Analysis

Values are expressed as means ± SD. We used one-way factorial analysis of variance followed by Duncan’s multiple range test for comparisons of group means or Student’s t test for two-group comparisons. A two-way ANOVA was performed to test the interaction between TSA and quercetin on cell growth, apoptosis, and the expression of p53 and acetyl histone in A549 cells. Differences were considered statistically significant at *p*<0.05. In addition, we determined the combination index in apoptosis and the expression of p53 and acetyl histone using the method described by Mai et al. [10] to confirm the nature of the combined effect of TSA and quercetin. The combination index is calculated as the ratio of observed value/expected; a value>1 indicates a synergistic effect, while a ratio<1 indicates a less than additive or an antagonistic effect. Whereas, the expected value of combined treatment is calculated as [(observed TSA-treatment value/control value) × (observed quercetin-treatment value/control value)] × (control value).

## Results

### Quercetin has Different Effects on TSA-induced Cell Growth Arrest and Apoptosis in A549 Cells and in H1299 Cells

**Figure 1:**
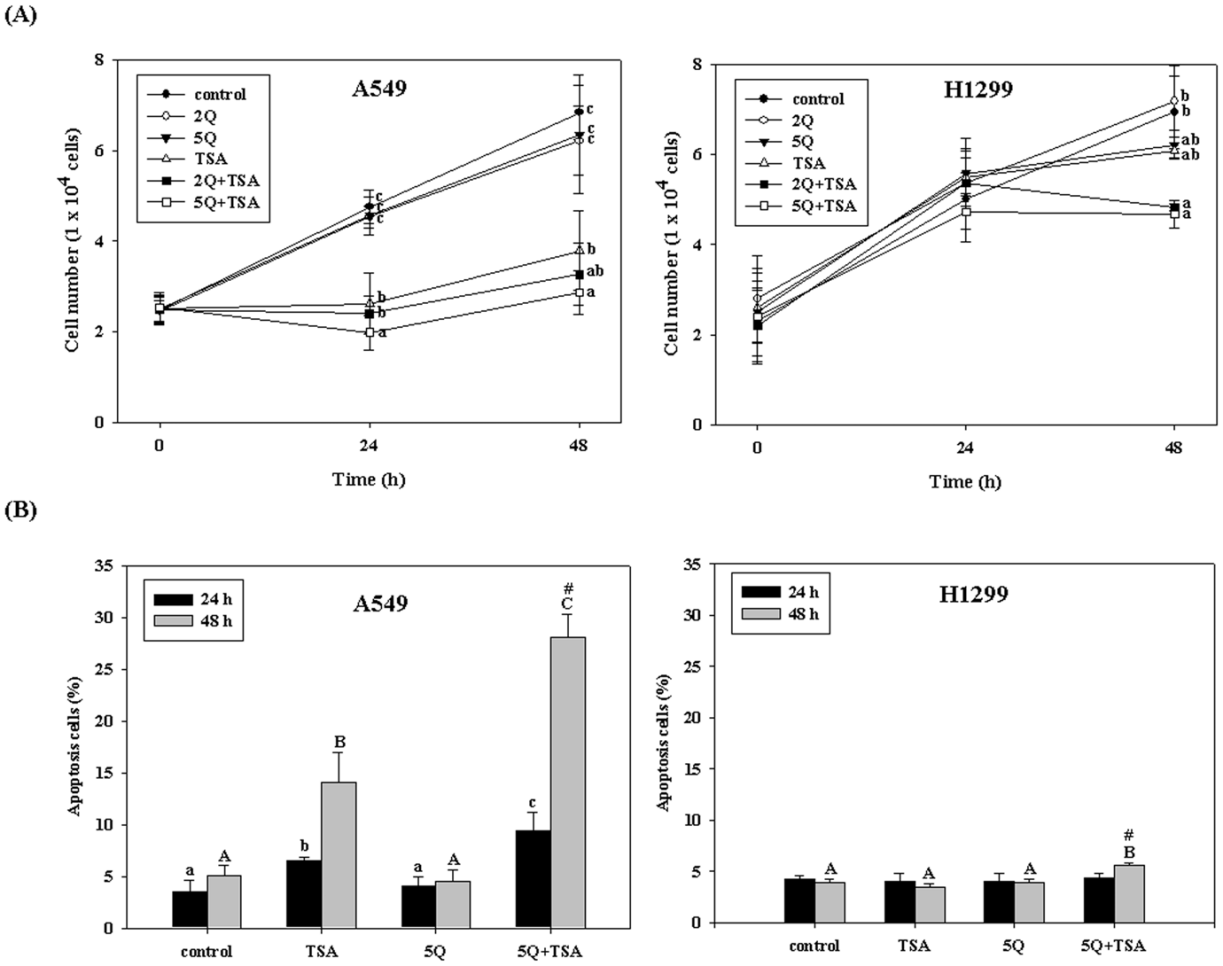
Effects of trichostatin A (TSA) alone or in combination with quercetin on the growth (A) and apoptosis (B) of A549 cells and H1299 cells. The cells were incubated with TSA (25 ng/mL) alone or in combination with 2 or 5 µM quercetin (2Q and 5Q, respectively) for 24 h and 48 h. Values (means ± SD, n = 3) at the same time not sharing a common letter (a-c and A-C for 24 and 48 h, respectively) are significantly different (*p*<0.05). The growth of H1299 cells at 24 h among groups was not significantly different. #denotes a significant interaction between TSA and quercetin (two-way ANOVA, *p*<0.05).

TSA significantly inhibited the growth of A549 cells (Fig. 1A), and quercetin significantly enhanced the cell-growth-arrest effect of TSA in a dose-dependent manner. Quercetin at a concentration of 5 µM significantly increased the suppressing effect of TSA by about 25% at both 24 h and 48 h. In H1299 cells, TSA alone slightly rather than significantly (*p*<0.05) induced cell growth arrest at 48 h, and the addition of quercetin only slightly increased the effect of TSA. Quercetin alone did not significantly affect cell growth at 24 and 48 h (Fig. 1A), however, it induced cell-growth-arrest at 72 h by about 15% (*p*<0.05) in both A549 and H1299 cells. Furthermore, we determined the enhancing effect of quercetin (5 µM) on TSA-induced apoptosis in both cell lines. Quercetin significantly and markedly increased TSA-induced apoptosis in A549 cells. The apoptosis level changed from 13% to 28% at 48 h. Even though quercetin also increased the TSA-induced apoptosis in H1299 cells, the apoptosis level only changed from 4% to 6% (Fig. 1B). The combined effect of TSA and quercetin on apoptosis at 48 h in both A549 and H1299 cells was synergistic (two-way ANOVA, *p<*0.001 and *p = *0.006, respectively; combination index = 2.0 and 1.6, respectively). Quercetin alone did not induce apoptosis even at 72 h (data not shown).

### Quercetin Increases p53 Protein Expression in A549 Cells

**Figure 2:**
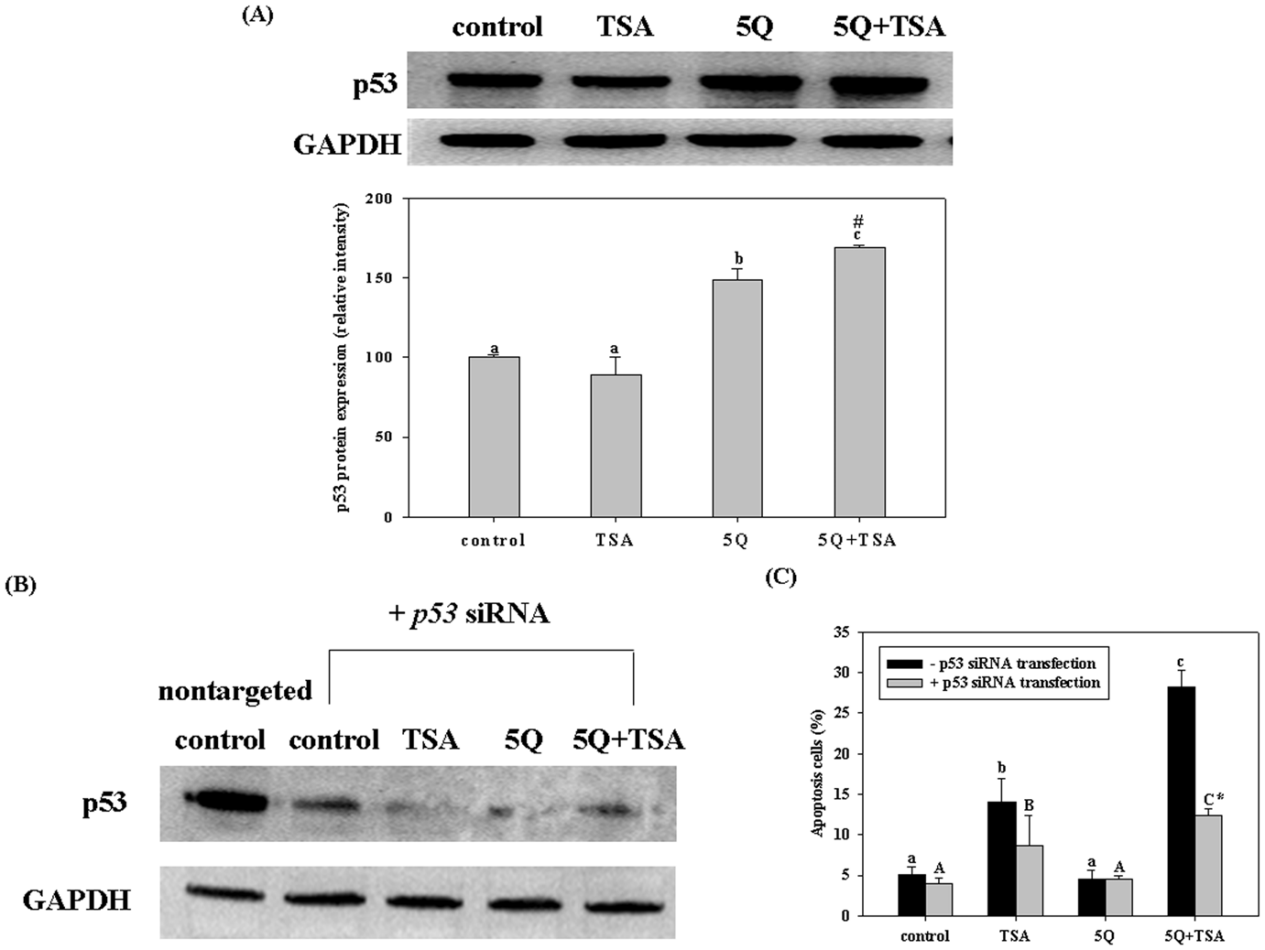
p53 protein expression in A549 cells without (A) or with (B) p53 siRNA transfection and the effect of p53 siRNA transfection on apoptosis (C) induced by various treatments. The A549 cells were transfected with or without p53 siRNA before incubation with 25 ng/mL trichostatin A (TSA) alone or in combination with 5 µM quercetin (5Q) for 12 h or 48 h for the p53 expression assay and apoptosis assay, respectively. Values (mean ± SD) in the same group not sharing a common letter (a-c and A-C for without or with p53 siRNA, respectively) are significantly different (*p*<0.05). *denotes a significant different from p53-normal cells in the same treatment. (*p*<0.05). #denotes a significant interaction between TSA and quercetin (two-way ANOVA, *p*<0.05).

A549 cells express wild-type p53 protein, whereas H1299 cells are a p53 null mutant. To investigate whether p53 plays a role in the different effects of quercetin in the different cell lines, we determined the expression of p53 in A549 cells exposed to TSA alone or in combination with 5 µM of quercetin. As shown in Fig. 2A, after incubation for 12 h, TSA alone did not increase the expression of p53 protein; quercetin alone increased p53 expression by 48% compared with the control group; whereas TSA in combination with quercetin significantly increased p53 expression by 69%. The combined effect of TSA and quercetin was synergistic (two-way ANOVA, *p* = 0.003; combination index = 1.3). To confirm the role of p53 in the enhancing effect of quercetin on TSA-induced apoptosis, we blocked p53 protein expression by transfection of p53 siRNA into A549 cells. The results showed that p53 siRNA markedly reduced the protein expression of p53 in A549 cells (Fig. 2B). Furthermore, the enhancing effect of quercetin on TSA-induced apoptosis at 24 h (data not shown) and 48 h was significantly diminished by transfection with p53 siRNA (Fig. 2C). However, p53 silencing did not completely inhibit the enhancing effect of quercetin on TSA-induced apoptosis in A549 cells, suggesting that the p53-independent pathway also contributes to the combined effect.

### Quercetin Enhances TSA-induced Apoptosis through the Mitochondrial Pathway in A549 Cells

**Figure 3:**
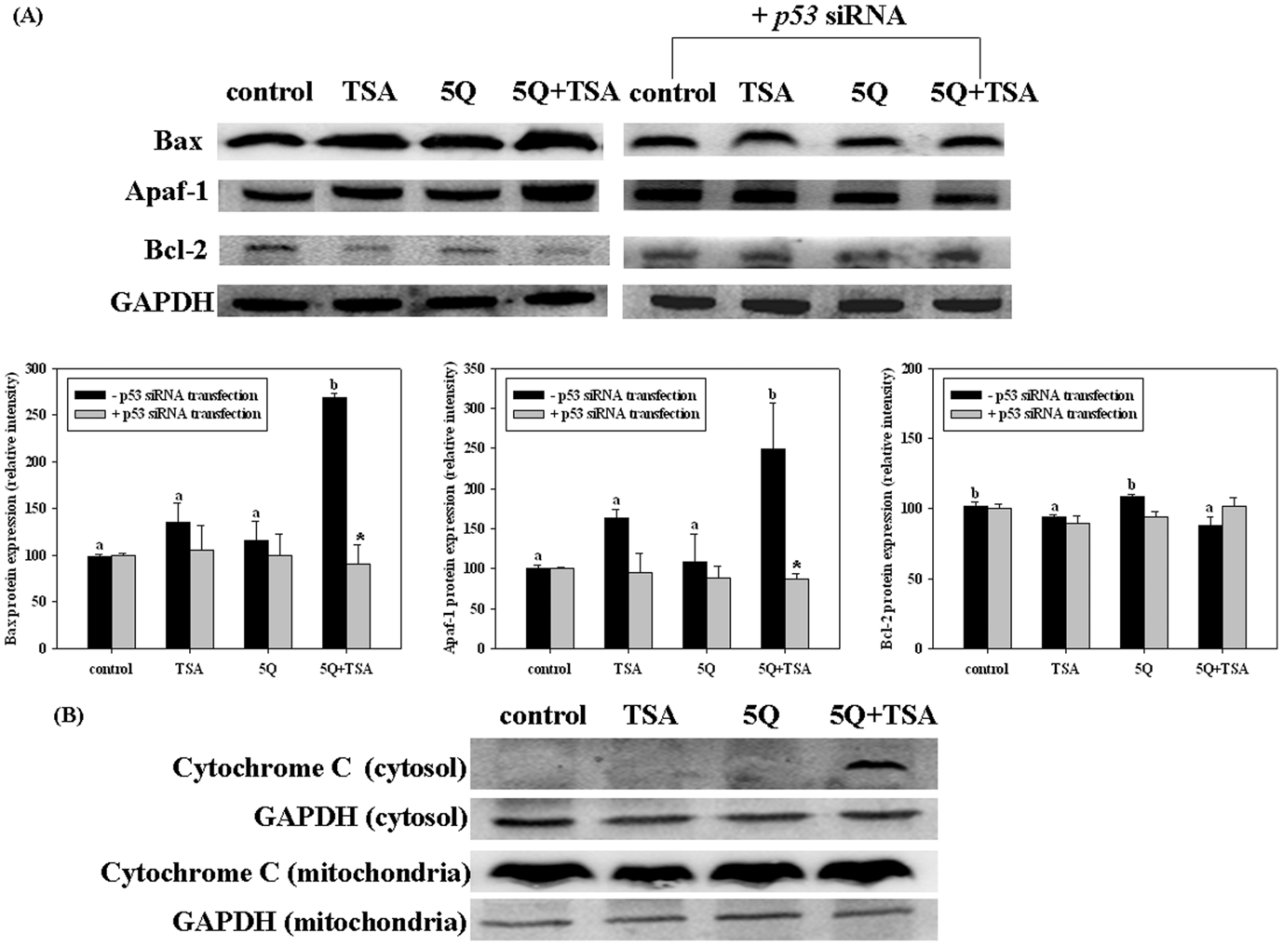
Effects of trichostatin A (TSA) alone or in combination with quercetin on Bax, Apaf-1 and Bcl-2 protein expression in A 549 cell without or with p53-silencing (A); cytochrome c levels in cytosol and mitochondria in A549 cells without p53-silencing (B). The A549 cells were transfected with or without p53 siRNA before incubation with TSA (25 ng/mL) alone or in combination with 5 µM quercetin (5Q) for 18 h. Values (means ± SD, n = 3) among the groups without p53-silencing not sharing a common letter (a-c) are significantly different (*p*<0.05). Values in p53-silenced cells with asterisks (*) are significantly different from in p53-normal cells with the same treatment (*p*<0.05).

To study the pathway contributing to the enhancing effect of quercetin on TSA-induced apoptosis in A549 cells, we performed a preliminary microarray assay after A549 cells were treated for 48 h. We found that compared to TSA alone, TSA in combination with quercetin increased the expression of several mitochondria-associated pro-apoptosis genes including Apaf-1 and caspase-9; whereas Bcl-2 expression in the groups with TSA alone or in combination with quercetin exposure were lower than that in the control group (data not shown). Further Western blot assay in cells with various treatments for 18 h confirmed this finding. As shown in Fig. 3A, TSA in combination with quercetin rather than TSA alone significantly increased the levels of Bax and Apaf-1 proteins compared with those in the control group. TSA alone and in combination with quercetin similarly suppressed the levels of Bcl-2 protein compared with the control group. p53 siRNA transfection suppressed TSA+quercetin -induced Bax and Apaf-1 protein expression (Fig. 3A). The effect of p53 siRNA on TSA alone or TSA+quercetin induced Bcl-2 expression was not significant. Furthermore, TSA in combination with quercetin rather than TSA alone markedly induced the release of cytochrome c into the cytosol after treatment for 24 h (Fig. 3B).

**Figure 4:**
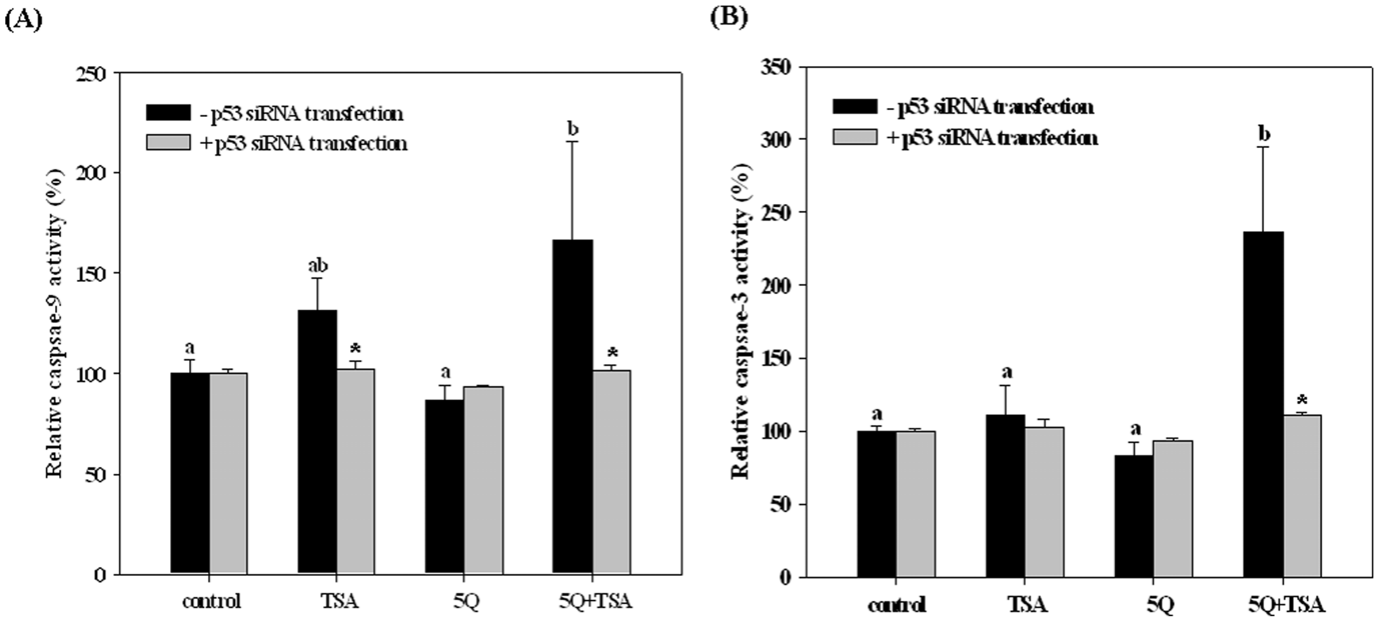
Effects of trichostatin A (TSA) alone or in combination with quercetin on caspase-9 (A) and caspase-3 activity (B) in A549 cells with or without p53-silencing. The A549 cells were transfected with or without p53 siRNA before incubation with 25 ng/mL TSA alone or in combination with 5 µM quercetin (5Q) for 24 h. Values (means ± SD, n = 3) among the groups without p53-silencing not sharing a common letter (a-c) are significantly different (*p*<0.05). Values in p53-silenced cells with asterisks (*) are significantly different from in p53-normal cells with the same treatment (*p*<0.05).

In addition, we used ELISA kits to determine the activities of caspase-9 and caspase-3 in A549 cells with various treatments for 24 h. Consistent with the above results, TSA slightly rather than significantly increased caspase-9 and caspase-3 activities, whereas quercetin significantly enhanced the TSA-induced activation of caspase-9 and caspase-3 by 27% and 113%, respectively (Fig. 4). Silencing p53 expression also suppressed the enhancing effect of quercetin on the TSA-induced activation of caspase-9 and caspase-3. These data indicated quercetin+TSA induced apoptosis at least in part through the mitochondria apoptosis pathway.

### Quercetin Enhances TSA-induced Histone Acetylation

**Figure 5:**
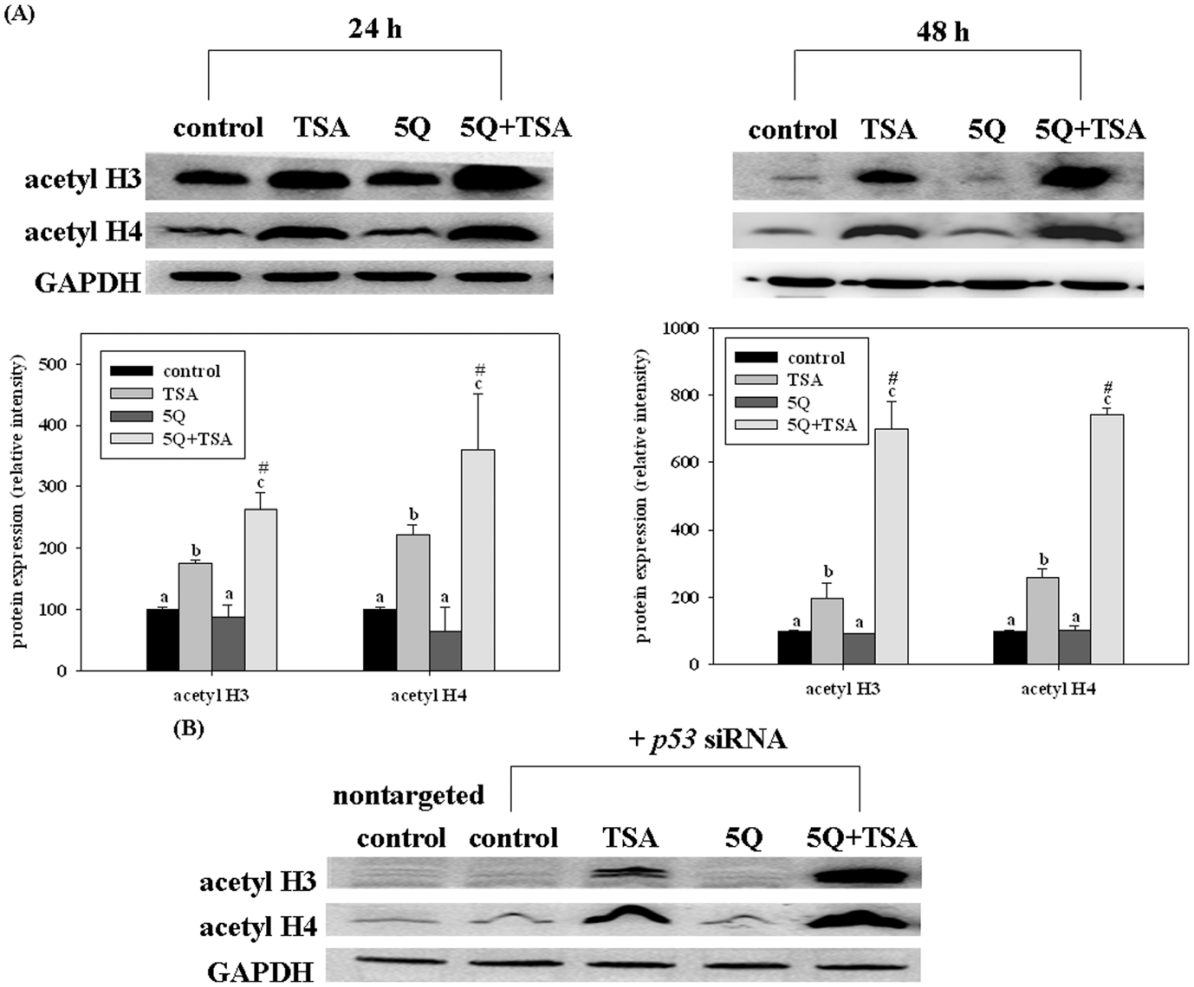
Effects of trichostatin A (TSA) alone or in combination with quercetin on the expression of acetyl histone H3 (acetyl H3) and H4 (acetyl H4) in A549 cells without (A) or with (B) p53 siRNA transfection. The cells were incubated with TSA (25 ng/mL) alone or in combination with 5 µM quercetin (5Q) for 24 or 48 h. Values (means ± SD, n = 3) not sharing a common letter (a-c) are significantly different (*p*<0.05). #denotes a significant interaction between TSA and quercetin (two-way ANOVA, *p*<0.05).

**Figure 6:**
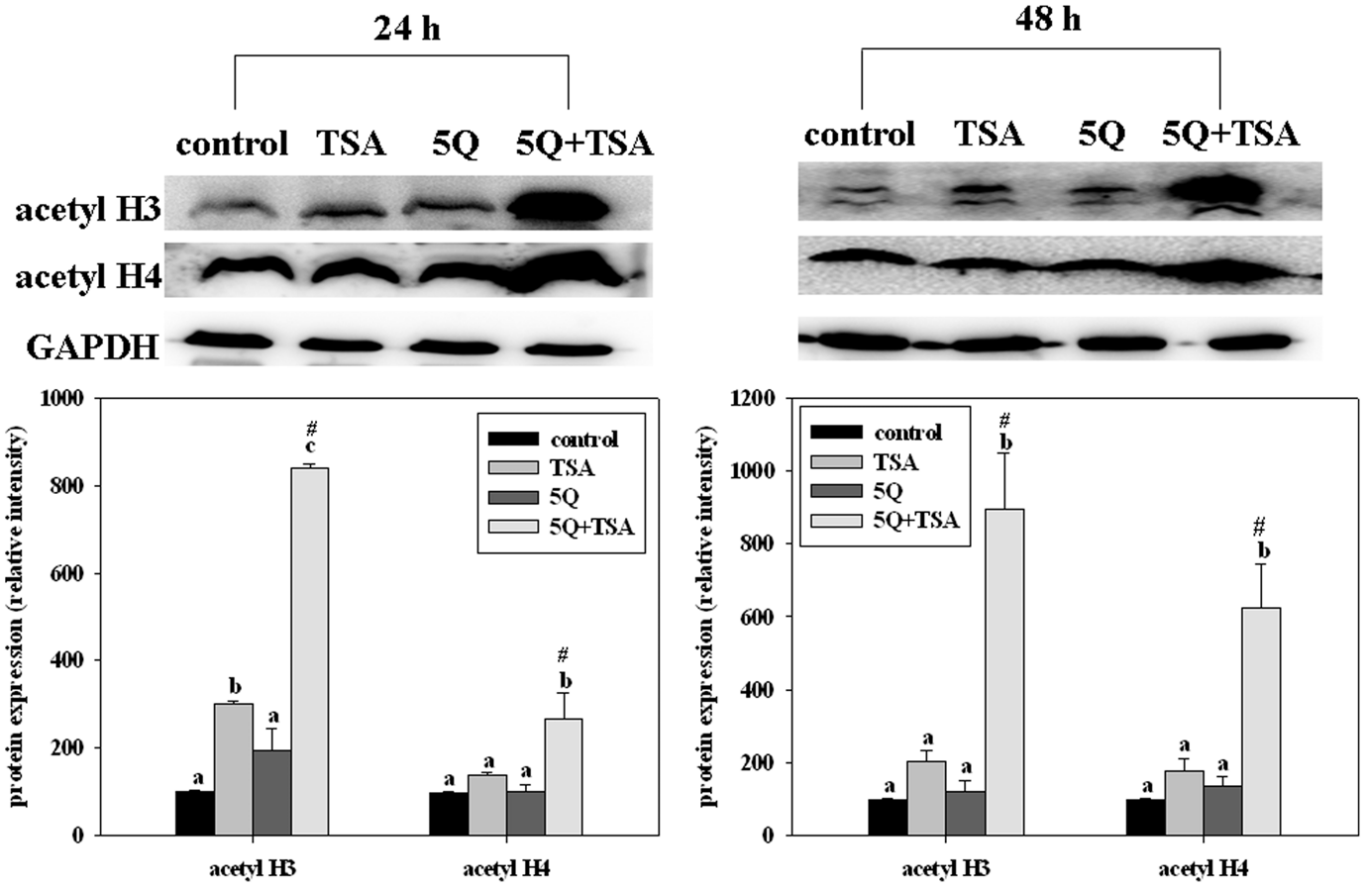
Effects of trichostatin A (TSA) alone or in combination with quercetin on the expression of acetyl histone H3 (acetyl H3) and H4 (acetyl H4) in H1299 cells. The cells were incubated with TSA (25 ng/mL) alone or in combination with 5 µM quercetin (5Q) for 24 or 48 h. Values (means ± SD, n = 3) not sharing a common letter (a-c) are significantly different (*p*<0.05). #denotes a significant interaction between TSA and quercetin (two-way ANOVA, *p*<0.05).

We investigated p53-independent pathways by which quercetin enhanced the effects of TSA since the p53-dependent pathway can not completely explain the combined effects on lung cancer cells. Because an increase in histone acetylation is one of the major mechanisms by which TSA induces apoptosis in various cancer cells [2], [3], we determined the effect of quercetin on TSA-induced acetylation of histones. The results showed that TSA alone significantly increased the acetylation of histones H3 and H4 at 24 h and 48 h (Fig. 5). Quercetin significantly enhanced the TSA-induced acetylation of histones H3 and H4 by about 50%–250% at 24 h and 48 h (Fig. 5A). The enhancing effect of quercetin was synergistic (two-way ANOVA, *p<*0.05; the combination index = 1.4 and 3.0 for histone H3; 1.5 and 2.8 for histone H4 at 24 and 48 h, respectively). In addition, silencing *p53* expression did not affect the enhancing effect of quercetin on TSA-induced histone acetylation (Fig. 5B). Furthermore, we found that the effects of TSA alone or in combination with quercetin on the expression of acetylated histones H3 and H4 in H1299 cells were similar to those in A549 cells, that is, quercetin also enhanced TSA-induced histones H3 and H4 acetylation in H1299 cells (Fig. 6). The combined effect was synergistic (two-way ANOVA, *p<*0.05; the combination index = 2.5 and 3.5 for histone H3; 1.4 and 2.7 for histone H4 at 24 and 48 h, respectively). These findings suggest that quercetin enhanced TSA-induced histone acetylation by p53-independent mechanisms and this may contribute to the enhancing effect of quercetin on apoptosis.

### Quercetin Enhances the Anticancer Effect of TSA in the Xenograft Tumor Model

**Figure 7:**
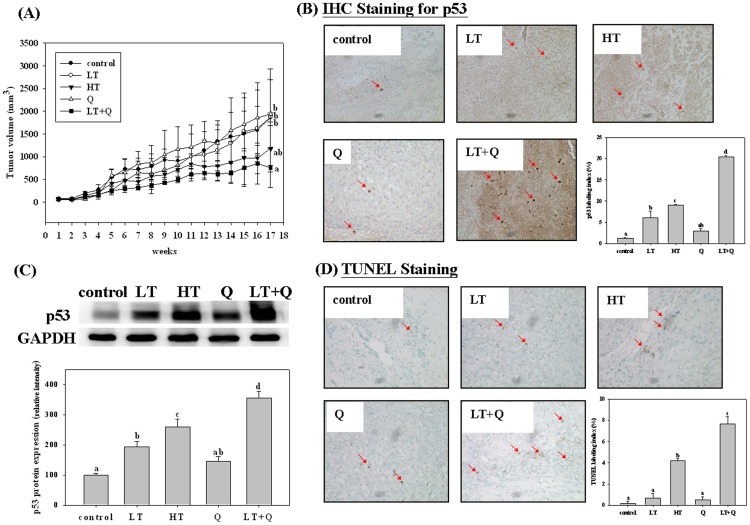
Effects of trichostatin A (TSA) alone or in combination with quercetin on tumor growth (A); p53 protein expression in tumor tissue determined by immunohistochemical (IHC) staining (B) and western blot (C); and the level of apoptosis in tumor tissue determined by TUNEL assay (D) in tumor-bearing mice. Thirty male nude mice at age 5 weeks were injected with A549 cells and after 4 weeks were treated for 13 weeks with TSA (low dose, 0.5 mg/kg body weight; high dose, 1 mg/kg body weight), or quercetin alone or combined as described in the Methods. The control group was administered the vehicle only. At the end of the experiment, the mice were sacrificed and tumor tissues were analyzed for p53 protein expression and apoptosis. Values (means ± SD, n = 6) not sharing a common letter (a-d) are significantly different (p<05). LT, low-dose TSA group; HT, high-dose TSA group; Q, quercetin alone group, LT+Q, low-dose TSA plus quercetin group.

To determine the potency of quercetin in enhancing the anticancer effect of TSA in vivo, we performed experiments with an A549 xenograft tumor model in nude mice. We found that a high dose (1 mg/kg body wt) of TSA was of borderline significance (t-test, *p* = 0.07) in inhibiting tumor growth in A549 tumor-bearing nude mice, whereas a low dose (0.5 mg/kg body wt) of TSA and quercetin alone had no effect (t-test, *p>*0.05). However, treatment with the low dose of TSA in combination with quercetin (administered through intraperitoneal injection) significantly inhibited tumor growth in A549 tumor-bearing nude mice (t-test, *p* = 0.0003, Fig. 7A). The antitumor effect of combined treatment was better than or similar to that of the high dose of TSA. In addition, histological assessments and Western blot assay also indicated that quercetin increased low-dose TSA-induced p53 expression in tumor tissue (Fig. 7B and C). Similar to our in vitro study, quercetin slightly increased the expression of p53. The level of apoptosis in tumor tissue from the combined treatment group was higher than in tissue from the control group or the group treated with each single compound (Fig. 7D).

## Discussion

Growing evidence suggests that the combination of phytochemicals with anticancer drugs may be a strategy for cancer therapy [10], [26], [27]. However, little has been reported about the combined effect of quercetin and TSA, except that quercetin in combination with TSA cooperatively induces cell death in human leukemia cells [20]. Similarly, in the present study, we first demonstrated that quercetin at a physiological dose (2 or 5 µM) enhanced TSA (25 ng/mL)-induced cell growth inhibition and apoptosis in A549 cells. Quercetin also increased the antitumor effect of TSA in H1299 cells at 48 h, however, the enhancing effect of quercetin on A549 cells (containing the wild type p53 gene) was markedly stronger than on H1299 cells (p53 null mutant). Compared to the TSA alone group, the increase in apoptosis induced by TSA in combination with quercetin seemed more marked than the decrease in cell growth (Fig. 1). This is due to apoptosis being expressed as the percentage of total cells. The percentage was low in the control and TSA alone group. Thus, the change induced by TSA+quercetin seemed marked. In fact, the increased numbers of apoptotic cells were lower than the decreased number in cell growth, suggesting that except for apoptosis, the inhibition of cell growth induced by TSA in combination with quercetin could also be due to the cell-growth-arrest effect or other modes of death.

The difference in sensitivity of A549 cells and H1299 cells to combined treatment suggests an association of p53 protein with the different effects of the combined treatment. The p53 protein, which is a known potent tumor suppressor, is maintained at low levels in unstressed cells [28]. In stimulated cells, p53 is increased and acts as a transcription factor to upregulate the expression of many genes, including cell-growth-arrest and apoptosis associated genes [29]. Several studies [17], [21], [30] have shown that quercetin itself suppresses cell growth and induces apoptosis in cancer cells. Consistent with these findings, in the present study, we found that quercetin significantly increased p53 expression in A549 cells whether they were exposed to TSA or not. However, TSA+quercetin significantly induced apoptosis after 24 h while quercetin only induced cell-growth-arrest up to 72 h. This may be due to the differences in p53 expression levels, the qualitative status of p53, and other cellular contexts, which have been suggested to influence p53 to stimulate cell cycle arrest or apoptosis [31], [32]. Furthermore, transfection of p53 siRNA into A549 cells markedly diminished the enhancing effect of quercetin on TSA-induced apoptosis, indicating that a p53-dependent pathway plays an important role in the enhancing effect of quercetin on TSA-induced apoptosis. The precise mechanisms by which quercetin increased p53 expression in the present study remain unclear. However, a study performed on melanoma cells showed that quercetin induces phase II detoxification enzymes, nicotinamide adenine dinucleotide phosphate:quinine oxidoreductase 1, which in turn stabilize p53 from ubiquitination [33]. Tanigawa et al. [21] also found that quercetin stabilizes p53 protein expression by inhibiting p53 ubiquitination in HepG2 cells. The authors found that quercetin also stabilizes p53 mRNA. In addition, genotoxicity of quercetin has also been shown to induce the increase of p53 expression [34]. However, we did not find quercetin at 5 µM induced DNA damage (data not shown), suggesting that this reaction may not contribute to the effect of quercetin in the present study.

It has been reported that quercetin acts through the mitochondrial pathway to induce apoptosis in cancer cells at high doses of 80 µM [17], [21]. Herein, we demonstrated that the mitochondria-mediated pathway also contributed to the enhancing effect of quercetin at a low dose on TSA-induced apoptosis in A549 cells. Our data showed that TSA in combination with quercetin increased Apaf-1 and Bax protein expression compared with that of the TSA alone group or the control group as well as the release of cytochrome c from mitochondria to the cytosol, which in turn increased the activation of caspase-9 and caspase-3. However, the combined treatment did not affect the expression of Bcl-2 compared with TSA alone. The Bax protein, a member of the Bcl-2 family, can promote apoptosis through the induction of cytochrome c release from the mitochondria [35], whereas the Apaf-1 protein is the structural core of the apoptosome, which binds and activates caspase-9 [36]. Both Bax and Apaf-1 are p53 target genes [36]–[38]. We also demonstrated that after silencing p53 expression, the increase in Bax and Apaf-1 proteins induced by TSA+quercetin in A549 cells was diminished. This evidence supports our conclusion that quercetin exerts its enhancing effect on the antitumor effect of TSA at least in part through a p53-dependent pathway.

Furthermore, using A549 tumor-bearing nude mice, we demonstrated that quercetin administered through intraperitoneal injection also enhanced the anticancer effect of TSA (0.5 mg/kg body wt) in vivo. The efficiency of the combined treatment was better than or similar to that of the high dose of TSA (1 mg/kg body wt). Quercetin also increased TSA-induced p53 expression and apoptosis in tumor tissue, which suggests that p53 may also play an important role in the enhancing effect of quercetin in vivo. A recent study showed that 50 mg/kg of quercetin (given by intraperitoneal injection 3 times) enhanced the antitumor effect of cisplatin in combination with gemcitabine [39] in vitro and in vivo. The study indicated that the suppression of Hsp27 expression, which is induced by chemotherapy and enhances the chemoresistance of lung cancer cells, contributes to the enhancing effect of quercetin. These findings and ours suggest that quercetin has potential as an adjuvant to chemotherapy and may reduce the dose of anticancer drugs and thereby reduce the toxicity of chemotherapy [11].

It has been demonstrated that the anticancer effect of TSA in lung cancer cells is associated with the induction of p53 protein and both death receptor- and mitochondria-mediated apoptosis pathways [4], [40]. Studies have shown that TSA at 100 nM up-regulates the ratio of Bax/Bcl-2, the activity of caspse-9, and the release of cytochrome c, three major components of the mitochondrial apoptosis pathway. However, we found that TSA at 25 ng/mL (82.5 nM) did not affect p53 protein expression, the ratio of Bax/Bcl-2, caspase-9 activity and the release of cytochrome c. This lack of effect may have been due to the lower dose and short incubation time we used because in our preliminary study we found that 25 ng/mL of TSA alone did not increase the expression of p53 protein, which induces apoptosis typically followed mitochondria pathway [41], until 48 h of incubation (data not shown). Our data suggests that TSA at 25 ng/ml induced apoptosis in A549 firstly through death-receptor pathway (or others) rather than mitochondria pathway.

Except for the p53-dependent pathway, p53-independent pathways could also be involved in the mechanisms underlying the enhancing effect of quercetin in both A549 and H1299 cells. Although the precise mechanisms are unclear, our results showed that quercetin increased the TSA-induced acetylation of histones H3 and H4 in A549 cells with or without p53 expression as well as in H1299 cells, p53 null cells, suggesting that this pathway may contribute to the p53-independent mechanisms. Studies have shown that histone deacetylase inhibitors induce histone acetylation, which in turn lead to cell apoptosis by p53-independent mechanisms [42]–[44]. For example, histone acetylation of specificity protein 1 binding sites on the p21 promoter, an important cell cycle and apoptosis-associated gene [38], [45], induces apoptosis; histone acetylation increases chromatin relaxation and enhances the accessibility of DNA to apoptotic endonucleases [46]. Our study also showed that TSA alone induced apoptosis of A549 cells at 24 h accompanied by an increase in acetylation of histones H3 and H4 rather than p53 protein expression. Thus, we postulate that histone acetylation may also account for the enhancing effect of quercetin on TSA-induced apoptosis in A549 cells. In agreement with our hypothesis, a recent study showed that histone hyperacetylation is involved in the human leukemia cell death induced by quercetin (75–100 µM) [47]. The precise p53-independent mechanisms associated with the effects of quercetin warrant further investigation.

In conclusion, our in vitro study demonstrated that quercetin enhanced the TSA-induced apoptosis in human lung cancer cells through p53-dependent and p53-independent pathways. We also confirmed the enhancing effect of quercetin in tumor-bearing mice. These findings suggest that the combination of quercetin and trichostatin A may be a potential strategy for cancer therapy.
